# Depletion of Sirtuin 1 (SIRT1) Leads to Epigenetic Modifications of Telomerase (TERT) Gene in Hepatocellular Carcinoma Cells

**DOI:** 10.1371/journal.pone.0084931

**Published:** 2014-01-08

**Authors:** Bin Zhang, Juan Chen, Alfred S. L. Cheng, Ben C. B. Ko

**Affiliations:** 1 Departments of Anatomical and Cellular Pathology, The Chinese University of Hong Kong, Hong Kong, China; 2 The Key Laboratory of Molecular Biology on Infectious Diseases, Ministry of Education, The Second Affiliated Hospital of Chongqing Medical University, Chongqing Medical University, Chongqing, China; 3 Institute of Digestive diseases, The Chinese University of Hong Kong, Hong Kong, China; 4 The Hong Kong Polytechnic University Shenzhen Research Institute, Hong Kong, China; 5 Department of Applied Biology and Chemical Technology, The Hong Kong Polytechnic University, Hong Kong, China; The University of Hong Kong, Hong Kong

## Abstract

Sirtuin 1 (SIRT1) is a nicotinamide adenine dinucleotide (NAD)-dependent deacetylase that is implicated in plethora of biological processes, including metabolism, aging, stress response, and tumorigenesis. Telomerase (TERT) is essential for telomere maintenance. Activation of TERT is considered a crucial step in tumorigenesis, and therefore it is a potential therapeutic target against cancer. We have recently found that SIRT1 expression is highly elevated in hepatocellular carcinoma, and the depletion of SIRT1 leads to substantial reduction in TERT mRNA and protein expression. However, the underlying molecular mechanism of SIRT1-dependent TERT expression remains uncharacterized. Here, we elucidated if SIRT1 regulates TERT expression via transcriptional, epigenetic and post-transcriptional mechanisms. We report that depletion of SIRT1 does not lead to significant change in transcriptional activity and CpG methylation patterns of the TERT promoter, nor does it affect mRNA stability or 3′-UTR regulation of TERT. Intriguingly, depletion of SIRT1 is associated with substantial induction of acetylated histone H3-K9 and reduction of trimethyl H3-K9 at the TERT gene, which are known to be associated with gene activation. Our data revealed that SIRT1 regulates histone acetylation and methylation at the TERT promoter. We postulated that SIRT1 may regulate TERT expression via long-range interaction, or via yet unidentified histone modifications.

## Introduction

Mammalian sirtuins are a family of NAD^+^-dependent enzymes that are homologous to the yeast silent information regulator 2 (SIR2) [1]. The yeast SIR2 regulates aging by maintaining transcriptional silencing of the mating-type loci, the ribosomal DNA locus and the telomeres. There are seven sirtuin members in human (SIRT1-7), each exhibits distinctive subcellular localization, enzymatic activities and functions [2]. These sirtuins are characterized by a conserved catalytic core domain flanked by a unique N- and/or C-terminal domain [3]. Among others, SIRT1 is a nucleocytoplasmic protein but predominantly localized to the nucleus [3]. It interacts with a variety of signaling molecules and nuclear factors in the cytoplasm and nucleus respectively, and plays a key role in energy metabolism, telomeric maintenance and genomic stability [4]–[7].

The functional role of SIRT1 in cancer is controversial, for it may act as a tumor promoter or suppressor depending on tumor type [8]. Nevertheless, evidence from different laboratories has suggested that SIRT1 expression is elevated and associated with tumor growth in prostate tumor and hepatocellular carcinoma respectively [9]–[13]. Earlier we showed that SIRT1 expression is up-regulated in HCC, and that the depletion of SIRT1 significantly inhibits proliferation of HCC cells via induction of cellular senescence or apoptosis [9]. We further demonstrated that depletion of SIRT1 is associated with substantial reduction of telomerase (TERT) mRNA and protein expression [9]. Given the pivotal role of TERT in tumorigenesis, we sought to further delineate the underlying mechanism of SIRT1-regulated TERT expression. Because depletion of SIRT1 has led to concomitant reduction of TERT mRNA and protein abundance, we hypothesized that SIRT1 might regulate TERT expression via transcriptional, epigenetic, or miRNA regulation. In this study we attempted to explore if SIRT1 regulates TERT expression via these regulatory mechanisms. We found that depletion of SIRT1 does not lead to significant change in transcriptional activity and CpG methylation patterns of the TERT promoter, nor does it affect mRNA stability or 3′-UTR regulation of TERT. Intriguingly, depletion of SIRT1 is associated with substantial induction of acetylated histone H3-K9 and reduction of trimethyl H3-K9 at the TERT gene, which are known to be associated with gene activation. Our data suggests that SIRT1 may regulate TERT expression via long-range interaction, or via yet unidentified histone modifications.

## Materials and Methods

### Cell Lines and Cell Culture

SK-HEP-1, SNU-423, PLC5 and Hep3B cells were obtained from American Type Culture Collection. SK-HEP-1, Hep3B and PLC5 cells were cultured in Dulbecco's modified Eagle's medium containing 10% FBS (Gibco BRL). SNU-423 cells were cultured in RPMI 1640 supplemented with 10% FBS. HepG2 cells were cultured in MEM with 10% FBS. All cells were maintained in a humidified incubator at 37°C with 5% CO2.

### Plasmids, antibodies, shRNAs and siRNAs

SIRT1 (04-1091), H3K9Ac (17-658), H3K9me3 (17-625), H3K4me2 (07-030), H3K27me3 (#07-449) antibodies were purchased from Millipore; TERT (sc-7212) antibodies were purchased from Santa Cruz Biotechnology; β-actin antibodies (A5316) were purchased from Sigma-Aldrich. Lipofectamine 2000 was from Life Technologies. Polybrene (H9268) and Actinomycin D (A1410) were purchased from Sigma-Aldrich.

Lentivirus plasmid vectors pLKO.1-puro vectors containing shRNA targeting SIRT1 (TRCN0000018980 and TRCN0000018981) were described previously [9]. pLKO.1-puro lentiviral vector expressing a non-targeting shRNA was used as a control. Promoterless (pGL3 basic), SV40 promoter-driven (pGL3-SV40) and pRL-TK luciferase reporter vector were purchased from Promega (Madison, WI). pMIR-REPORT luciferase vector was purchased from Life Technologies. The pGL3-1000 and pGL3-400 reporter vectors were constructed by subcloning the 1.0-kb and 0.4-kb fragment of the TERT proximal promoter into the Xho I and Hind III sites of pGL3 basic luciferase reporter vector respectively. pGL3-400/ex1 vector was constructed by subcloning the 0.4-kb promoter fragment and exon 1 of TERT gene into the Xho I and Hind III of pGL3 basic luciferase reporter vector. pMIR-TERT 3′UTR was generated by inserting the 554-bp 3′UTR of TERT gene into the Hind III and Spe I sites of pMIR-REPORT luciferase vector. The sequences of all the constructs were verified by DNA sequence determination.

### DNA transfections and viral transductions

For luciferase reporter assays, cells were plated to a 24-well plate at a density of 3×10^4^ per well at 24 hours prior to transfection experiments. Cells were co-transfected by luciferase reporter plasmid and pRL-TK reporter plasmid using Lipofectamine 2000. pGL3-SV40 was used as a control. For lentiviral transduction, cells were transduced with appropriated amount of lentivirus for 12–15 hours in the presence of polybrene (8 µg/mL). Then the supernant was removed and replaced with fresh medium. The cells were cultured for 4–5 days before harvesting for experiments.

### RNA Extraction, Reverse Transcription, and Real-time PCR

Total RNAs were extracted from cells by using Trizol Reagent (Invitrogen) according to manufacturer's instruction. Genomic DNA was removed by DNase I digestion. cDNA was synthesized by using PrimeScript™ RT Master Mix kit (TaKaRa). Real-time quantitive PCR (RT-PCR) assays was conducted using the SYBR® Premix Ex Taq™ II kit (TaKaRa). The PCR experiments were performed with ABI PRISM 7900 Fast Real-Time PCR System. Primers used in real-time PCR experiments were: TERT (forward 5′-GCGTTTGGTGGATGATTTCT-3′ and reverse 5′-CAGGGCCTCGTCTTCTACAG-3′), β-actin (forward 5′- CTGGCACCACACCTTCTACAATG-3′ and reverse 5′-AATGTCACGCACGATTTCCCGC-3′).

### Western blotting analysis

Cells were harvested and lysed by using RIPA lysis buffer (50 mM Tris-HCl, pH 7.4, 150 mM NaCl, 0.5% sodium deoxycholate, 1% Nonidet P-40, 0.1% SDS, 1 mM EDTA, 2 mM sodiumorthovanadate, 50 mM sodium fluoride, 10 µg/ml aprotinin, 10 µg/ml leupeptin, 1 mM PMSF) supplemented with Protease Inhibitor Cocktail (Roche). Protein concentration was determined using Protein Assay Kit (BioRad) with bovine serum albumin (Sigma) as the standard. Thirty μg protein samples were mixed with 5×SDS loading buffer (10% SDS, 50% glycerol, 0.01% bromophenol blue, 7% DTT, 50 mM Tris pH 6.8) and was resolved by SDS-polyacrylamide electrophoresis. The protein was transferred to a nitrocellulose membrane, and immunoblotted with antibodies as indicated. Blots were developed with ECL Western blotting reagents (Thermo Fisher Scientific).

### Bisulfite sequencing

The CpG methylation in TERT gene flanking the transcription start site was determined by using bisulfite sequencing analysis described previously [14]. Genomic DNA was purified by phenol-chloroform extraction. Bisulfite modification of genomic DNA was performed by using the EZ DNA Methylation-Gold Kit™ (Zymo Research Corp.) according to manufacturer protocol. CpG methylation was analyzed using three different primer sets spanning a region from −400 to +150 from the transcription start site of TERT. The sequences of primers are: BSP-1, Forward 5′-TTGTTTTTAGGGTTTTTATATTATGGT-3′, Reverse 5′- CAAAACTAAAAA ATAAAAAAACAAAAC-3′; BSP-2, Forward 5′- TAGTTTTGTTTTTTTATT TTTTAGTTT-3′, Reverse 5′- CCAACCCTAAAACCCCAAAC-3′. PCR products were separated on 1.0% agarose gels and purified using QIAquick Gel Extraction kit accroding to the manufacturer's instructions (Qiagen). Purified PCR products were cloned into pCR2.1-TA vector (Invitrogen) and transformed into bacteria. Plasmid DNA was isolated using QIAprep Spin Miniprep kit following the manufacturer's instructions (Qiagen). Plasmids DNA was determined uisng using DNA sequencing. For each reaction at least 8 colonies were sequenced. The methylation status of each CpG residue was determined and analysed using the QUMA online tools (http://quma.cdb.riken.jp/) [15].

### Quantitative chromatin immunoprecipitation (ChIP) assay

The ChIP assay was performed as previously described [16] with slight modification. SK-HEP-1 cells or HepG2 cells (1×10^7^) transduced with the indicated lentiviral shRNA were incubated in 10-cm dishes for 4 days. Cells were crosslinked for 15 min at room temperature using 1% formaldehyde, washed twice in ice cold PBS containing 1 mM PMSF, resuspended in 400 µl cell lysis buffer (50 mM Tris-Cl pH 8.0, 10 mM EDTA and 1% SDS, 1 mM PMSF and 1X protease inhibitor), and were kept on ice for 10 min. The chromatins were sheared by sonication using a Bioruptor (Diagenode Inc.) to generate DNA fragments ranging from 500–1000 bp. After centrifugation, the lysates were diluted 10-fold with ChIP dilution buffer (16.7 mM Tris-Cl pH 8.1, 2 mM EDTA, 167 mM NaCl, 0.01% SDS and 1.1% Triton X-100), and pre-cleared with Protein A Agarose/Salmon sperm DNA (Upstate Biotechnology) at 4°C for 30 min then immunoprecipitated overnight at 4°C with H3K9Ac, H3K9me3, H3K4me2, H3K27me3, and SIRT1 antibodies respectively. The antibody-chromatin complexes were then precipitated by Protein A Agarose/Salmon sperm DNA, washed, and eluted. DNA and proteins complexes were reverse crosslinked by heating at 65°C for 5 hours in the presence of 0.2 M NaCl, and digested with proteinase K buffer overnight at 56°C. Subsequently, the samples were purified by phenol-chloroform extraction and precipitated in ethanol overnight at - 20°C. The samples were centrifuged at 13,000 rpm for 10 min at 4°C, and the pellets were washed once using 70% ethanol and air-dried. The precipitated DNA was resuspended and was used for PCR. Each sample was performed in triplicates. RT-PCR anlaysis was carried out using ABI PRISM 7900 Fast Real-Time PCR System. Primer sets used for the assay of the TERT gene are as follows: 1) −869 bp to −739 bp: forward 5′- GGGATGTGACCA GATGTTGG-3′, reverse 5′- AGAAAGGGTGGGAAATGGAG-3′; 2) −33 bp to +111 bp: forward 5′-AGCCCCTCCCCTTCCTTTCC-3′, reverse 5′- AGCGCACGGCTCGGCAGC-3′; 3) +787 bp to +888 bp: forward 5′- CTGGAACCATAGCGTCAGG-3′, reverse 5′- TTGGGCAACGGCAGACTT-3′. Occupancy of each of the histone or SIRT1 protein was normalized to input, and expressed as the fold difference relative to shCont-expressing cells.

### Analysis of mRNA stability

The stability of TERT mRNA was determined as described [17], [18]. SK-HEP-1 cells expressing shCont or shSIRT1-1 were treated with 5 µg/ml of Actinomycin D to inhibit mRNA synthesis. Total RNA was extracted at the indicated time points, and the remaining endogenous TERT and β-actin mRNA levels were analyzed by real-time RT-PCR. The amount of TERT mRNA remaining was quantified by real-time RT-PCR and normalized to the amount of β-actin mRNA.

### Statistical analysis

Statistical analysis was performed using the Student *t*-test (two-tailed), or Mann-Whitney U-test as indicated. All data were expressed as mean +/−SD.

## Results

**Figure 1:**
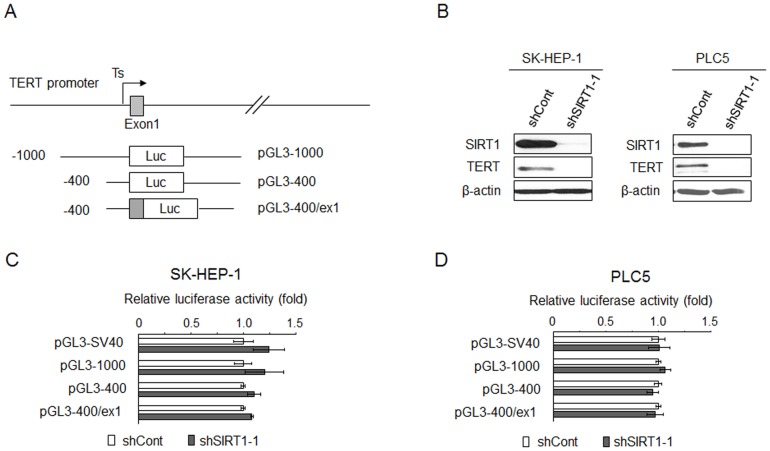
Depletion of SIRT1 did not affect TERT promoter activity. (A) A schematic representation of TERT promoter reporter plasmids. The TERT promoter reporter plasmids were generated by inserting the 1.0 kb and 0.4 kb DNA fragments of TERT promoter upstream of the initiating ATG or 0.4 kb core promoter as well as the first exon of TERT into luciferase (Luc) reporter vector pGL3-Basic in the sense orientation. Arrow, the transcription start site. Numbers, the number of bases upstream (-) and downstream (+) of the translational start codon. (B) Western blot shows the TERT reduction following SIRT1 suppression in SK-HEP-1 and PLC5 cell lines. (C) and (D) Sub-confluent cultures of SK-HEP-1 or PLC5 cells expressing the indicated shRNA in 24-well plate were transfected with indicated luciferase reporter plasmids along with a pRL-TK reporter plasmid as a control for transfection efficiency. The pGL3-SV40 containing the minimal, enhancerless SV40 promoter was used as a control. After 48 h of incubation, cells were lysed and the lysates were analyzed for dual luciferase activity. Normalized relative luciferase activity in shCont-expressing cells was designated as 1.0. Results are the mean+/−S.D. of triplicate measurements from one of three representative experiments with similar results. Statistical analysis was performed with Student's t-test.

Evidence suggested that SIRT1 could enhance the expression of PGC-1α gene by forming a transcriptional complex with MyoD [19]. It also interacts with LXR to positively regulate the expression of LXR target genes [20]. Intriguingly, however, the SIRT1-MyoD complex functions as negative regulator for myogenin gene expression [21]. These data suggested that SIRT1 may directly regulate gene transcription. To elucidate the role of SIRT1 in transcriptional regulation of TERT expression, we cloned a 1000 bp (pGL3-1000) and 400 bp (pGL3-400) human TERT promoter DNA into luciferase reporter respectively. Because earlier findings also suggested that the proximal exonic region of TERT gene may play a role in the regulation of promoter activity [22], a reporter construct that was driven by the 400 bp promoter DNA together with exon 1 (pGL3-400/ex1) was also generated (Figure 1A). To explore the role of SIRT1 in the regulation of TERT promoter activity, SK-HEP-1 cells were first transduced with lentivirus expressing shRNA against SIRT1 (shSIRT1-1) or non-targeting shRNA (shCont) respectively, and were subsequently co-transfected with each of the luciferase reporter and renilla reporter. TERT mRNA (not shown) and protein (Figure 1B) expression was successfully depleted by the transduction of shSIRT1-1. Nevertheless, the luciferase activity of pGL3-1000, pGL3-400, and pGL3-400/ex1 was not altered in SK-HEP-1 cells depleted for SIRT1 (Figure 1C). Similar observations were made when PLC5 cells were used for the analysis (Figure 1D). These data suggested that SIRT1 does not regulate TERT expression via transcriptional regulation at the proximal promoter.

**Figure 2:**
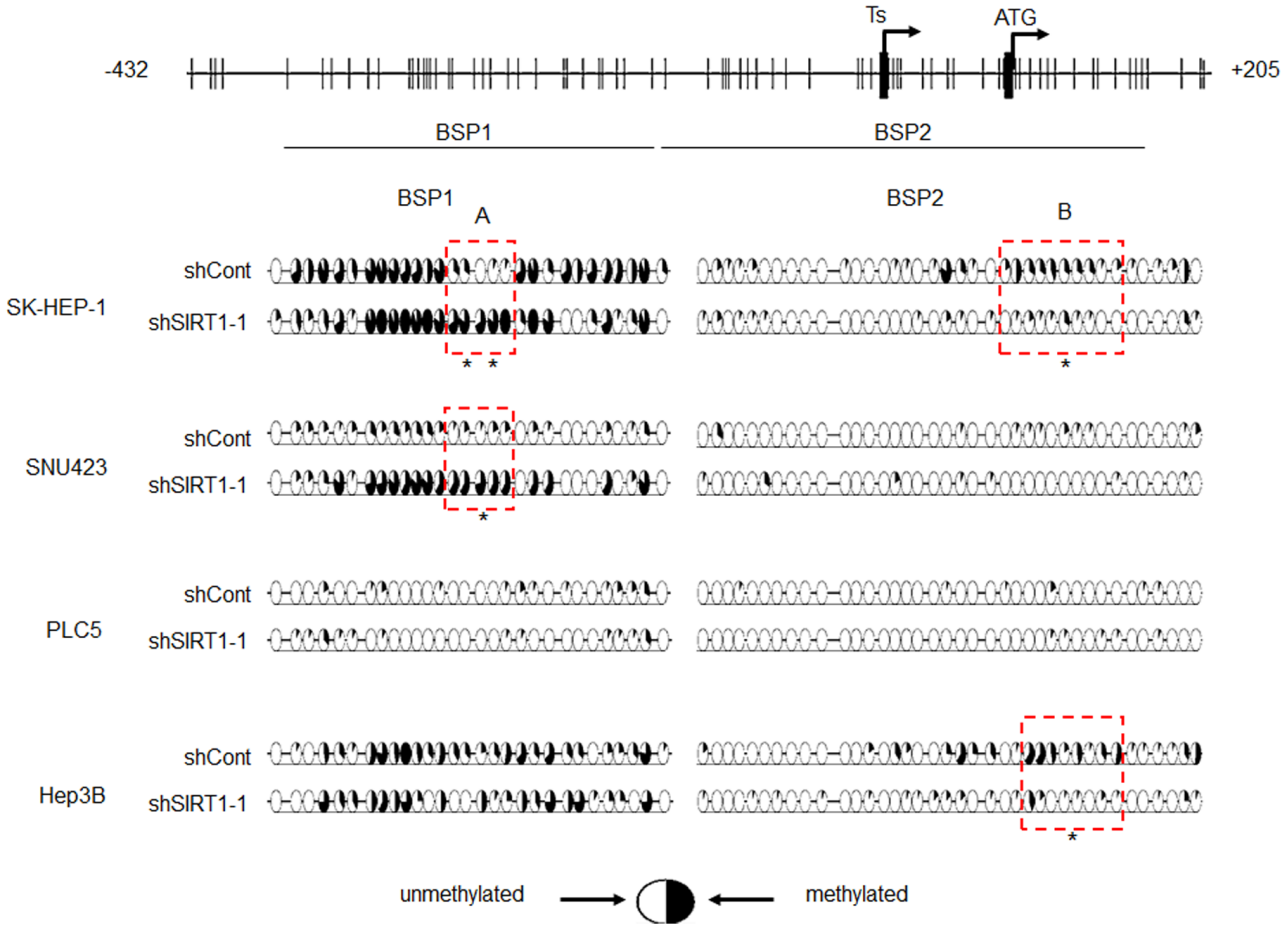
Bisulfite sequencing of TERT gene in different HCC cell lines following SIRT1 depletion. Bisulfite-modified genomic DNAs of HCC cell lines expressing the indicated shRNA was amplified by PCR using two independent primer sets (BSP-1 and BSP-2) spanning the proximal promoter region of TERT. PCR products were cloned into pCR2.1-TA vectors, and at least 8 clones were sequenced and analyzed for their methylation status by using the QUMA online tools (http://quma.cdb.riken.jp/). The percentage of methylated clones at each CpG site is depicted by a pie chart. The number of the pie charts corresponds to the number of the CpG positions on the map. In SK-HEP-1 and SNU423 cells, SIRT1-depletion enhanced CpG methylation between −266 to −240 bp upstream of TERT transcription start site. In contrast, SIRT1-depletion reduced CpG methylation between +74 to +122 bp in SK-HEP-1 cells and between +82 to +122 bp in Hep3B cells. * P<0.05, ** P<0.01 versus lentiviral control shRNA-transduced cells by the Mann-Whitney U-test.

CpG sites are present in the regulatory region of TERT gene [23]. We therefore explored if SIRT1 regulates TERT expression by altering CpG methylation. The methylation profile of CpG sites flanking the transcriptional start site of TERT gene (-400 bp to +150 bp relative to the transcription start site) was determined in several HCC cell lines using bisulfite sequencing analysis. We found variable CpG methylation pattern in different HCC cell lines. While CpG sites 5′ to the transcriptional start sites are substantially methylated in SK-HEP-1 and Hep3B cells, it is only modestly methylated in PLC5 and SNU423 cells (Figure 2). In SK-HEP-1 cells, expression of shSIRT1-1 enhanced CpG methylation at a region 5′ (region A) to the transcriptional start site (TSS), while it diminished CpG methylation at a region 3′ (region B) to the TSS. In Hep3B cells, shSIRT1-1 only reduced CpG methylation at region B; whereas in SNU423 cells, it only enhanced methylation at region A. Furthermore, the expression of shSIRT1-1 did not alter CpG methylation pattern in PLC5 cells (Figure 2). TERT expression was consistently repressed by SIRT1 silencing in these HCC cell lines [9]. Therefore, the inconsistent alternation in CpG methylation pattern in response to SIRT1 depletion suggested that SIRT1 might not regulate TERT expression via epigenetic control of CpG methylation.

**Figure 3:**
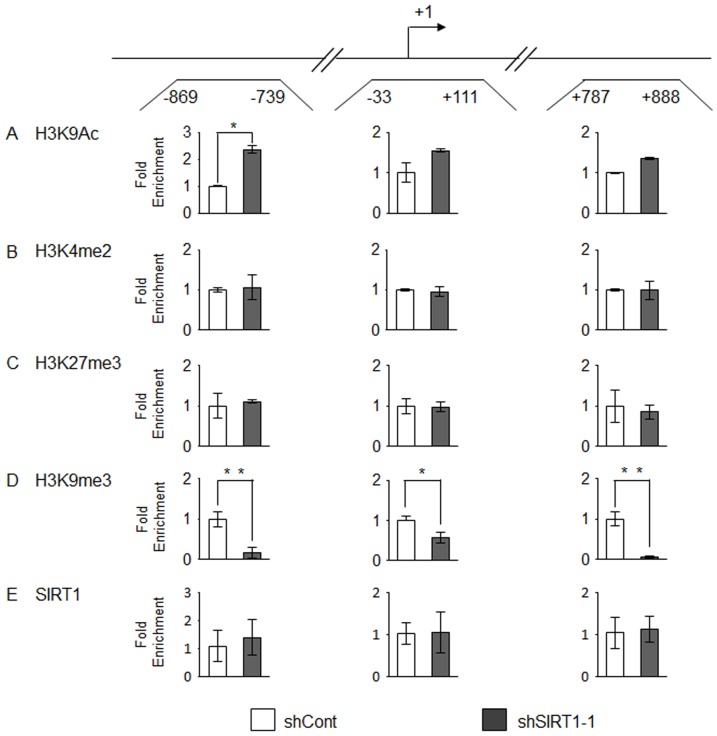
Alterations of histone modification and of SIRT1 occupancy on TERT gene following SIRT1 depletion in SK-HEP-1 cells. Three regions (shown in the upper panel) were investigated by ChIP-qPCR analysis. Arrow represents the transcription start site. Occupancy of each of the chromatin marks: acetyl-H3K9 (H3K9Ac), dimethyl-H3K4 (H3K4me2), trimethyl-H3K27 (H3K27me3) and trimethyl-H3K9 (H3K9me3) as well as SIRT1 protein are normalized to input and shown as the fold difference relative to that in shCont-expressing cells. Each sample was analyzed in triplicate in qPCR. All results are representative of one of three independent experiments with similar results. * P<0.05, ** P<0.01 versus lentiviral control shRNA-transduced cells by Student's t-test.

Earlier studies have discovered the role of epigenetic regulations in TERT expression. Specifically, increased acetylation of H3-K9, as well as the dimethylation of H3-K4 at around the transcriptional start site are known to positively regulate TERT expression [14], [24]. To explore if depletion of SIRT1 is associated with a reduction of these active chromatin marks, we determined post-translational modification of histones in SK-Hep-1 cells by chromatin immunoprecipitation (ChIP) assay followed by quantitative PCR analysis of three amplicons spanning the distal 5′ regulatory region (−869 to −739 bp), transcriptional start site (−33 to +111 bp), and gene body (+787 to +888 bp) of TERT gene respectively. Unexpectedly, depletion of SIRT1 led to a significant enrichment of acetylated histone H3-K9 in the distal 5′ regulatory region (Figure 3A). In contrary, change in the level of dimethyl H3-K4 was negligible in all three loci (Figure 3B). To further explore if SIRT1 depletion might enhance the level of other known inactive chromatin marks around TERT gene, leading to transcriptional repression, we measured the level of trimethyl H3-K27 and trimethyl H3-K9 respectively. The level of trimethyl H3-K27 was not altered by SIRT1 depletion (Figure 3C). However, intriguingly, there was a profound reduction in trimethyl H3-K9 across all three loci (Figure 3D). Similarly, in HepG2 cells, SIRT1 gene silencing also led to a significant enrichment of acetylated histone H3-K9 across all three loci. In these cells, there was also a remarkable reduction of the trimethyl H3-K9 in the gene body (+787 to +888 bp) (Figure S1). These findings are at odds with the established role of these histone marks in the repression of gene expression. Finally, to determine if SIRT1 is associated with TERT gene, we conducted ChIP analysis of SIRT1 in shSIRT1-1 and shCont cells respectively. Our data suggested that SIRT1 signal at TERT gene was not differ between cells expressing shSIRT1-1 and shCont (Figure 3E), suggesting that SIRT1 did not directly associated with TERT gene, at least in proximity to the TSS, to regulate its transcription.

**Figure 4:**
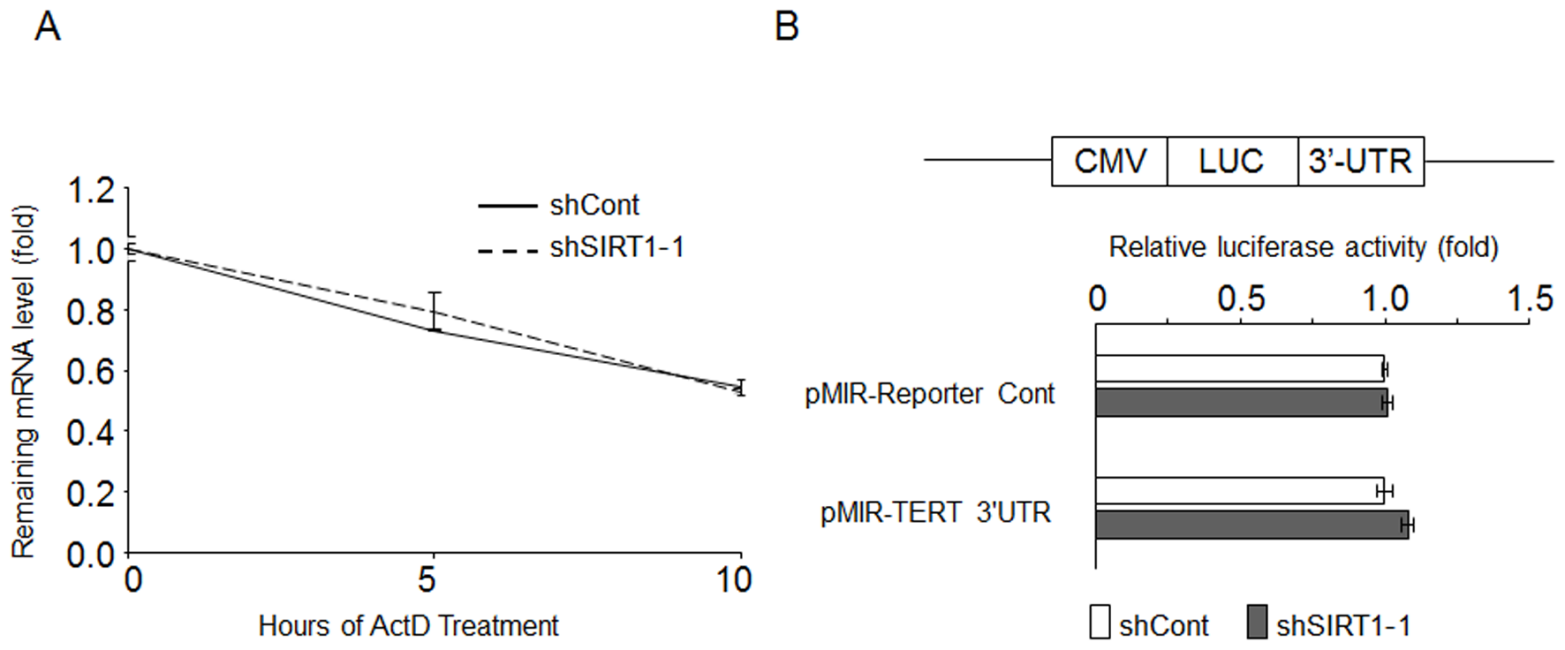
mRNA stability and microRNA may not play a role in TERT regulation by SIRT1. (A) SK-HEP-1 cells expressing the indicated shRNA were treated with 5 µg/ml of Actinomycin D for different duration. The remaining TERT mRNA level was determined by using qRT-PCR analysis by triplicates. The ratio of TERT to β-actin in each sample was calculated and then the amount of TERT mRNA remaining at indicated time points relative to time zero was determined. The graphs shown are representative of three individual experiments with similar results. (B) Dual luciferase reporter assay of TERT 3′UTR activity in SK-HEP-1 cells. Two days after lentiviral shRNA transductions, the pMir-Reporter vector only or the construct containing full-length TERT 3′UTR were transiently cotransfected along with pRL-TK reporter plasmid. Normalized relative luciferase activity in shCont- expressing cells was designated as 1.0. Results are the mean+/- S.D. of triplicate measurements from one of three representative experiments with similar results. Statistical analysis was performed with Student's t-test.

Besides transcriptional regulation, the expression level of TERT mRNA and protein could potentially be regulated by post-translational mechanisms. Among others, mRNA stability and translational efficiency could be regulated by miRNA in a 3′-untranslated region (3-UTR)-dependent manner. To this end, we first determined if SIRT1 regulates the stability of TERT mRNA. SK-HEP-1 cells were first transduced with lentivirus expressing shCont and shSIRT1-1 respectively. After 2 days, actinomycin D chase experiments were performed with these cells respectively. The degradation of TERT mRNA was virtually identical in cells expressing shCont and shSIRT1-1, suggesting that the stability of TERT mRNA was not regulated by SIRT1 (Figure 4A). On the other hand, to determine if SIRT1 regulates the function of 3′UTR, TERT 3′UTR luciferase reporter (pMir-TERT 3′UTR) was transfected into SK-HEP-1 cells expressing shSIRT1-1 and shCont respectively, followed by measuring the reporter gene activity. As shown in Figure 4B, compared to shCont expressing cells, cells that express shSIRT1-1 did not show a reduction in TERT 3′UTR luciferase activity. Together these data suggested that SIRT1 does not regulate TERT expression via the 3′UTR.

## Discussion

Previously we showed that SIRT1 expression is upregulated in a subset of HCC, and its overexpression is associated with more advance tumor grades [9]. Our finding was further corroborated by two other studies from Korea and Taiwan respectively [10], [11]. A more recent study further demonstrated that sirtuin inhibitor may delay the growth of human HCC tumor in an orthotopic xenograft model [25], suggesting that blocking the activity of sirtuins might be a novel treatment strategy for HCC. Therefore a thorough understanding of how SIRT1 regulates tumor progression will provide crucial information for bringing sirtuin inhibitors into the clinical use in the future.

Our earlier study suggested that the depletion of SIRT1 is associated with reduced expression of PTOP and TERT respectively, whereas ectopic expression of each of these genes significantly restored cell proliferation in SIRT1-depleted cells [9]. PTOP is a member of the shelterin complex that protects the telomeres from being recognized as sites of DNA damages [26], whereas TERT is essential for telomere maintenance and tumorigenesis [27]. In this study we sought to elucidate the underlying mechanism of SIRT1-regulated TERT expression. Because SIRT1 has been shown to regulate transcriptional activity, either by recruited directly to the promoter [28], [29] or indirectly by modifying the activity of other transcription factors [30], we therefore determined if SIRT1 may exert its effect via regulating the transcriptional activity of the TERT promoter. Our data suggested that SIRT1 did not regulate TERT expression via modulating the transcriptional activity of the 1 kb TERT promoter and the proximal exonic region. However, we could not rule out the possibility that SIRT1 might control TERT transcription via acting at a more distal regulatory region or via long-range interaction.

SIRT1 has also been shown to regulate gene expression via CpG methylation [29], [31], [32] and histone modifications [33], [34]. We therefore determined if SIRT1 regulates TERT expression via these epigenetic mechanisms. Although depletion of SIRT1 leads to modest change in CpG methylation pattern in different HCC cell lines, the inconsistent change among the cell lines studied suggested that SIRT1 might not regulate TERT expression via CpG methylation. In fact, evidence regarding the role of CpG methylation in TERT expression is subjected to debate. Earlier studies suggested that methylation of the TERT promoter is associated with gene silencing [35]. Enhanced CpG methylation of TERT promoter has been also associated with TERT activation [36]. Furthermore, there are evidence suggesting that the absence of methylation at and around the transcriptional start site of TERT is essential for its expression [14]. Taken together, although CpG methylation pattern was altered in the absence of SIRT1, our data did not provide convincing evidence that SIRT1 regulates TERT expression via CpG methylation. On the other hand, analysis of the regulatory region and gene body of the TERT gene revealed that depletion of SIRT1 is associated with substantial induction of H3-K9Ac at the proximal promoter and reduction H3-K9me3 respectively. It agrees well with the established functions of SIRT1, which has been known to directly deacetylate histone H3-K9 [33], and indirectly regulate H3-K9me3 level by targeting suppressor of variegation 3-9 homologue 1 (SUV39H1) [34]. H3-K9Ac and H3-K9me3 is associated with gene activation and inactivation respectively [37], and therefore heightened H3-K9Ac and reduced H3-K9me3 level would have led to an increase in TERT expression. Therefore, our finding that TERT expression was reduced is at odds with the current understanding of the roles of these histone modifications. Nevertheless, our data therefore confirmed that SIRT1 regulates the acetylation and methylation of H3-K9 at TERT promoter. We speculated that SIRT1 might regulate TERT expression via post-translational modification(s) of other histone residues, although the nature and position(s) of the modifications remains to be identified.

## Supporting Information

Figure S1 **Alterations of acetyl-H3K9 (H3K9Ac) and trimethyl-H3K9 (H3K9me3) occupancy on TERT gene following SIRT1 depletion in HepG2 cells.** Occupancy of the chromatin marks acetyl-H3K9 (H3K9Ac) and trimethyl-H3K9 (H3K9me3) were determined by ChIP-qPCR analysis and were shown as the fold difference relative to that in shCont-expressing cells. Each sample was analyzed in triplicate in qPCR. All results are representative of one of three independent experiments with similar results. * P<0.05, ** P<0.01 versus lentiviral control shRNA-transduced cells by Student's t-test.
(TIF)
