# Supplementary figures and images for: Depletion of Sirtuin 1 (SIRT1) Leads to Epigenetic Modifications of Telomerase (TERT) Gene in Hepatocellular Carcinoma Cells
Source: PLoS One. 2014 Jan 8;9(1):e84931. doi: 10.1371/journal.pone.0084931 (PMC3885646; doi:10.1371/journal.pone.0084931)

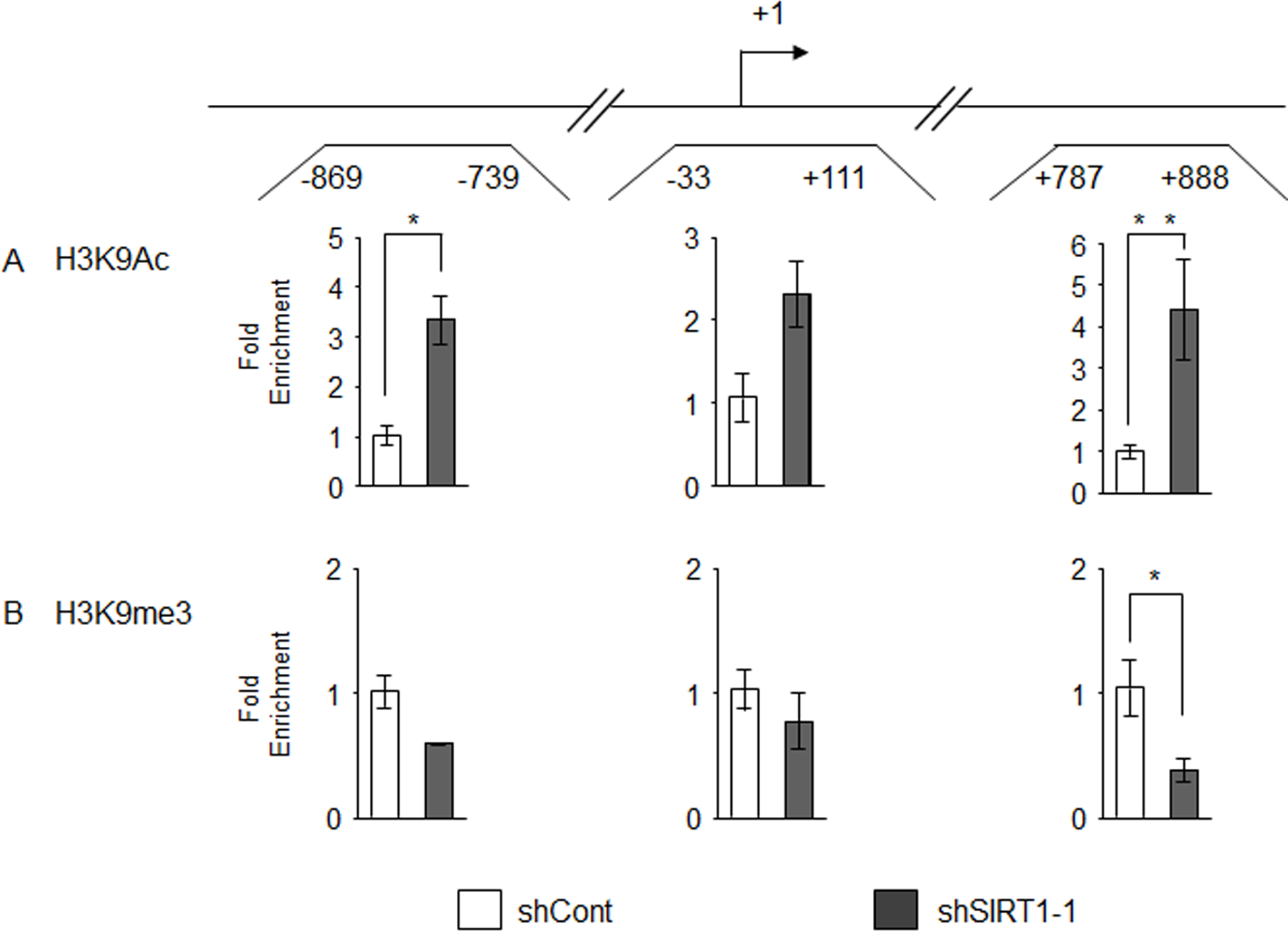

Supplement: Figure S1 — Alterations of acetyl-H3K9 (H3K9Ac) and trimethyl-H3K9 (H3K9me3) occupancy on TERT gene following SIRT1 depletion in HepG2 cells. Occupancy of the chromatin marks acetyl-H3K9 (H3K9Ac) and trimethyl-H3K9 (H3K9me3) were determined by ChIP-qPCR analysis and were shown as the fold difference relative to that in shCont-expressing cells. Each sample was analyzed in triplicate in qPCR. All results are representative of one of three independent experiments with similar results. * P<0.05, ** P<0.01 versus lentiviral control shRNA-transduced cells by Student's t-test. (TIF) [file pone.0084931.s001.tif]
